# Perspective of Parents of Infants and Children with Hearing Impairment about Hearing Aid Management: A Questionnaire-Based Study

**DOI:** 10.1055/s-0045-1810004

**Published:** 2025-09-10

**Authors:** Anil Kumar, Rashmi J. Bhat

**Affiliations:** 1 Department of Hearing Studies, Dr. S. R. Chandrasekhar Institute of Speech and Hearing, Bangalore, Karnataka, India

**Keywords:** hearing aid management, parent perspective, outcomes, pediatric audiology

## Abstract

**Introduction:**

It is important to understand the intricacies of hearing aid management early in the child's life to appreciate the full benefits of early intervention.

**Objective:**

Identifying aspects of hearing aid management that carers regard as taxing will allow hearing professionals to focus and encourage these areas, potentially leading to higher hearing aid use and better outcomes.

**Methods:**

49 parents of children with hearing impairment participated in the study. Parent Hearing Aid Management Inventory (PHAMI) was used to explore parent access to information and experiences in three domains such as parent skills and confidence in hearing aid management, hearing aid use, and communication with the audiologist using a 4-point rating scale. The data was collected using direct interviews and through Microsoft forms.

**Results:**

The majority of the parents desired information and support in identifying hearing aid options/accessories, obtaining financial assistance for costs, and using a listening stethoscope for determining when the hearing aid was not working. The most common challenges identified by parents as influencing their child's hearing aid use were activities (for example, playing, travelling in the car), and the fear of losing and damaging the hearing aids. Although many parents were pleased with the audiologist's assistance, they desired that the audiologist be more patient and contact them more regularly after the hearing aids were fitted.

**Conclusion:**

Professionals can help with effective device management by addressing parental challenges, collaborating on specific device management goals, and helping as needed.

## Introduction

It is well known that early and successful intervention for children with hearing impairment is essential. The timely detection of permanent hearing loss in infants and children is critical because early intervention services are needed to reach their full potential in these individuals. UNHS (Universal Newborn Hearing Screening) services provide early detection of hearing loss, allowing for early hearing aid fitting and specialized communication interventions. Early intervention with hearing aids or cochlear implants is linked to improved speech and language outcomes as well as improved long-term learning outcomes including reading cognisance. 1 2 3

Advancement in hearing technology (such as digital hearing aids and cochlear implants) and treatments that teach a child to learn spoken language through listening have made a significant difference in the lives of children with hearing loss. For audibility, regular hearing aid management is also important. Pediatric hearing aid management involves a comprehensive approach to ensuring that children with hearing loss receive the best possible support. Regular adjustments are crucial to accommodate the child's growth and changes in their hearing needs. Ongoing monitoring and support from audiologists, with family involvement, are key to ensuring the child's hearing aids effectively enhance their communication and developmental skills. Parents are responsible for ensuring that their children wear their hearing aids and that they are in working order. Higher language performance is associated with more frequent use of hearing aids and greater access to language input. 2 4 Children are in a crucial phase of language acquisition and using the hearing aid for less than 10 hours per day has been shown to have a negative impact on their language growth. 2

Parents have complained about a lack of training on how to verify aids and/or a lack of necessary equipment which can lead to irregular system monitoring. 5 6 Routine testing by hearing care professionals and parents is needed to ascertain the benefit of a hearing aid and to detect deficiencies in audibility that need to be addressed. Parents have indicated that different child and parent variables (e.g., child behavior, annoyance, and dejection) interfere with how frequently their children use their hearing aids, 7 8 9 and that average hours of use vary widely. 8 9 With data logging, the audiologist can see the average length of time the child wears the hearing aids. Emotional difficulties have been identified, including acknowledging the hearing loss, 5 appreciation of the benefits of hearing aid use, 10 parental mistrust in their specific skills (e.g., hearing aid listening check, troubleshooting problems, retention issues), and the expertise of other carers. 11

Given the difficulties parents have described, it is unsurprising that many parents have stated that these issues prevent their child from using hearing aids consistently. 5 9 10 There is also evidence to suggest that the use of hearing aids varies significantly depending on the listening environment, the age of the child, the severity of the hearing impairment, and the financial situation of the family. 6 9 12 The difficulties of effectively using and managing hearing aids at a young age should not be underestimated. 6 Caregivers may lack the necessary expertise for effective hearing aid management, or they may fail to apply their knowledge.

To fully appreciate the advantages of early intervention, it is necessary to recognise the complexities of hearing aid management at an early age. Identifying details of hearing aid management that carers report as difficult would enable audiologists to concentrate on focusing and promoting these aspects, potentially leading to increased hearing aid use and improved outcomes. This will assist in the development of behavior change interventions to promote hearing aid use and maintenance beginning with the initial prescription and fitting of the hearing aids. Using questionnaires designed to evaluate the experiences of both the child and their family with hearing devices helps hearing therapists understand the perceived benefits of these devices, as well as identify any potential factors that may affect the child and their careers. In 2015, Muñoz and colleagues used the Parent Hearing Aid Management Inventory (PHAMI) to explore the challenges caregivers face when managing hearing aids. They gathered participants from early intervention services in two different U.S. states. The inventory typically looks at aspects such as the frequency of hearing aid use, maintenance routines, and the impact of the device on the child's communication and quality of life. Therefore, this study aimed to investigate the challenges experienced by parents of infants and children with hearing impairment related to confidence in hearing aid management, challenges faced with the hearing aids, and communication with the audiologist from an Indian perspective.

## Methods

### Participants

Parents (mothers = 30 and fathers = 19) of 49 young children with hearing loss were recruited from the Department of Hearing Studies at Dr. S. R. Chandrasekhar Institute of Speech and Hearing. Ethical clearance was obtained from the institutional ethical committee for carrying out the proposed study. Informed written permission was taken from the parents by elucidating the aim and need of the study. Parents were provided with a copy of the consent and the right to withdraw from the study at any time.

### Materials

Parent Hearing Aid Management Inventory (PHAMI) developed by Munoz et al 2015 was used to explore parent access to information and experiences in three domains, such as parent skills and confidence in hearing aid management, hearing aid use, and communication with the audiologist. Caregivers had to answer four additional free-text questions to elaborate on the three main topics and share any additional thoughts they may have regarding their experiences with their child using hearing aids. Caregivers are also asked how often their child wore their hearing aid: *all waking hours, most of the day (over 5 hours), some of the day (1 to 5 hours), or hardly at all (less than 1 hour).* Scoring for the questions of all 3 domains was based on the 4-point rating scale. In domain 1, the scores indicated the need for information and training, where 1 indicated NO, got this information already, 2- NO, did not get this and do not want it, 3- YES, got this but I need more help, 4- YES, did not get this but I want help. In domain 2, the scores indicated problems that interfere with the child using his/her hearing aids, where 1 indicated never a problem, 2- Sometimes a problem, 3- frequently a problem, and 4- always a problem. In domain 3, scores indicated communication with the audiologist in learning and support needed to manage the child's hearing aids, where 1 indicated Yes, my needs are being met, 2- Yes, but I want this more often, 3- No, but I want this, 4- No, and I don't want this. There are four additional free-text questions.

### Procedures

Demographic data survey forms were provided to parents of the children who were fitted with hearing aids. Due to the challenges posed by the COVID-19 pandemic, we employed different tools for data collection to ensure safety, maintain social distancing, and adapt to the limitations of in-person interactions. The interview was carried out using a paper-pencil method. The investigator sat along with the parents and carried out the one-to-one interview with the respondents who were proficient in English. The questionnaire was also provided to a group of parents, and the questions were read out one by one with examples mentioned in the questionnaire. If parents encountered confusion, specific technical terms were translated into the native languages of Hindi or Kannada to ensure clarity and understanding. The questionnaire was also sent to some parents through social media as Microsoft forms which included the informed consent, demographic details, and the question sections. Microsoft form specified details regarding the study such as its purpose and its importance. It was also made sure that instructions regarding how to fill in the Microsoft form were explained in the simplest manner.

### Statistical Analysis

All the data obtained were tabulated using Microsoft Office-Excel 2019. Appropriate statistics were applied using the IBM Statistical Package for Social Sciences (SPSS) software version 20.0.

## Results

### Hearing Aid Management Information

Parents were inquired about the information they received and whether more information was required for each of the items on the questionnaire ( Table 1 ). Most of the parents (60-70%) reported receiving a lot of information about determining whether the hearing aids were benefitting the child, and about ways to prevent losing hearing aids. Information regarding options for hearing aids, accessories, and improving the child's hearing in noisy environments was only given to one parent. Many parents (55-70%) wanted more information about the hearing aid options, accessories, and about improving the child's hearing in noisy environments. None of the parents received information about obtaining loaner hearing aid and 87.7% of parents wanted more information about the same. Almost half of the parents wanted more information on finding financial assistance with only 18% receiving it. Although audiologists had given them information, it was either inadequate or of insufficient quantity, and many families wanted more details or assistance.

**Table 1:** Information Parents Received and Desired

Information	Received, No help needed % (n)	Not Received and help not needed % (n)	Received but would appreciate more help % (n)	Not received but will need help % (n)
1. How to determine if the hearing aids are helping	69.3(34)	0 (0)	28.5(14)	2(1)
2. How to prevent losing the hearing aids	61.2(30)	0 (0)	36.7(18)	2(1)
3. Meeting other parents with children with hearing loss	42.8(21)	2(1)	30.6(15)	24.4(12)
4. What child can/cannot hear ''without'' hearing aids	59.1(29)	0 (0)	32.6(16)	8.1(4)
5. Knowing about hearing aid options/accessories	2(1)	2(1)	40.8(20)	55.1(27)
6. What child can/cannot hear ''with'' hearing aids	59.1(29)	0 (0)	32.6(16)	8.1(4)
7. How to get loaner hearing aids	0 (0)	4(2)	8.1(4)	87.7(43)
8. Finding financial assistance	18.3(9)	0 (0)	34.6(17)	46.9(23)
9. Knowing when the audiologist needs to check the hearing aids	34.6(17)	2(1)	59.1(29)	4(2)
10. How to help the child hear better in noisy places	2(1)	0 (0)	26.5(13)	71.4(35)

### Hearing Aid Management Skills

Parents were asked about the skills and reported if they received training and if more help was desired for each of the items on the questionnaire ( Table 2 ). Most of the parents (60-80%) reported being taught each of the skills. However, few of them (20-30%) requested additional assistance with each of the skills listed. Just over half (55%) of the parents reported having not received any information on how to use a listening stethoscope to check hearing aid function but would appreciate it if they got any assistance on that. Parents need a lot of tools to monitor hearing aid functions. Results indicated only 3 parents had a listening stethoscope, 2 had a battery tester and almost all had (46) cleaning tools to clean their aids.

**Table 2:** Skill Parents Received and Desired

Skills	Received, No help needed % (n)	Not Received and help not needed % (n)	Received but would appreciate more help % (n)	Not received but will need help % (n)
1. When to change the hearing aid batteries	85.7(42)	2(1)	12.2(6)	0(0)
2. Clean earmolds and reattach tubing to the hearing aid	73.4(36)	2(1)	22.4(11)	2(1)
3. How to know when the child needs new earmolds	61.2(30)	2(1)	22.4(11)	14.2(7)
4. How to keep hearing aids on when child resists	65.3(32)	0 (0)	32.6(16)	2(1)
5. How to teach others to help manage the hearing aids	57.1(28)	4(2)	30.6(15)	8.1(4)
6. How to use listening stethoscope to check hearing aid function	16.3(8)	4(2)	24.4(12)	55.1(27)
7. How to do the Ling 6 sound test	83.6(41)	0 (0)	8.1(4)	8.1(4)
8. How to do basic hearing aid maintenance	63.2(31)	0 (0)	32.6(16)	4(2)

Parents were asked an open-ended question regarding having any other comments related to the information they received. Most of the parents wanted information on upgrading to a new hearing aid, dehumidifier box use, rechargeable batteries, and maintenance of the hearing aid. Parents should be counselled about various aspects related to information and skills with hearing aid management. Intensive training and counselling can help improve their knowledge and confidence in certain skills, making them adept at handling the hearing aid better.

### Hearing Aid Use Challenges

Parents were asked to rate 14 hearing aid using challenges on a never-to-always scale and to identify how frequently each item presented a challenge (see Table 3 ). The issues that parents faced on a regular basis were diverse.

**Table 3:** Hearing Aid Use Challenges

Challenges	Always % (n)	Frequently % (n)	Sometimes % (n)	Never % (n)
1. Distractions and needs of other children at home	0(0)	6(3)	34.6(17)	59.1(29)
2. Activities (playing outside, riding in the car)	4(2)	16.3(8)	67.3(33)	12.2(6)
3. The child not wanting to wear the hearing aids	6(3)	6(3)	34.6(17)	53(26)
4. Difficulties getting a set routine	2(1)	12.2(6)	28.5(14)	57.14(28)
5. The hearing aids not working	0(0)	0(0)	36.7(18)	63.2(31)
6. Other caregiver's ability to manage hearing aids	2(1)	12.2(6)	44.8(22)	40.8(20)
7. Costs	0(0)	2(1)	36.7(18)	61.2(30)
8. Concerns about how the hearing aids look	0(0)	6(3)	18.3(9)	75.5(37)
9. Not seeing my child benefiting from hearing aids	0(0)	8(4)	20.4(10)	71.4(35)
10. Frequent ear infections	0(0)	0(0)	24.4(12)	75.5(37)
11. Frequent feedback	0(0)	8(4)	38.7(19)	53(26)
12. Not being convinced that my child needs hearing aids	2(1)	2(1)	20.4(10)	75.5(37)
13. Pressure from others not to use the hearing aids	0(0)	12.2(6)	42.85(21)	44.8(22)
14. Fear of losing hearing aids	0(0)	12.2(6)	38.7(19)	48.9(24)

Parents faced a wide range of difficulties regularly; the most mentioned being activities (16.3%), difficulty getting a set routine (12.2%), other caregivers' ability to manage hearing aids (12.2%), pressure from others about using the hearing aids (12.2%), and fear of losing hearing aids (12.2%). About 8% of parents reported problems with frequent feedback and not seeing their child getting benefit from aids.

Parents were requested to estimate the number of hours their child normally used their hearing aids based on the child having a good or bad day. On good days, the child used hearing aids for all waking hours (30%, n =15); 8-9 hours (36.7%, n = 18); 5-7 hours (20.4%, n= 10); and5 hours (12.2%, n = 6). Parents indicated that their child used hearing aids during all waking hours (6%, n = 3); 8-9 hours (12.2%, n = 6); 5-7 hours (44.8%, n = 22); and,5 hours (36.7%, n = 18) on their bad days.

### Communication with the Audiologist

To determine whether the communication and assistance received from their audiologists suited their needs or if they desired additional involvement, parents were asked to consider about 13 topics related to communication with their audiologist (see Table 4 ).

**Table 4:** Communication with the Audiologist

Communication	Needs met % (n)	Needs more support % (n)
**1.** Ask for my thoughts and opinions	71.4(35)	28.57(14)
**2.** Respond to my input in a way that I feel understood	87.7(43)	12.3(6)
**3.** Be accepting of my challenges	87.7(43)	12.3(6)
**4.** Check in with me to see if I need help or support	44.89(22)	55.1(27)
**5.** Give me the opportunity to talk about how I am feeling	73.4(36)	26.6(13)
**6.** Help me recognize what I am doing right	55.1(27)	44.89(22)
**7.** Help me explore solutions to problems with hearing aids	44.8(22)	55.2(27)
**8.** Help me to monitor problems until the concern is resolved	51(25)	49(24)
**9.** Talk in a way I can understand	87.7(43)	12.3(6)
**10.** Help me gain confidence managing the hearing aids	81.6(40)	18.4(9)
**11.** Respect my culture and beliefs	85.7(42)	14.3(7)
**12.** Provide me with concrete resources	53(26)	47(23)
**13.** Teach me in ways I can learn	51(25)	49(24)

While most parents said their communication needs were being met, 55% (n = 27) said they would like more frequent check-ins with the audiologist to see if they need any support or assistance and help which allows them to explore solutions to problems with the aids. Parents were asked if the audiologist helped them recognize what they were doing right. 55.1% (n = 27) of parents were satisfied with their interaction with the audiologist; however, 44.89% (n = 22) of parents wanted more support. Parents were asked if the audiologist helped them monitor problems until the concern was resolved, 51% (n = 25) of parents reported their needs were met, although 49% (n = 24) parents wanted it more often. Almost 50% (n = 23) said they would like concrete resources (in writing and hands-on).

## Discussion

Regular hearing aid management is necessary for audibility. Parents must ensure that their children wear their hearing aids and that they are in good working order. Better language results are linked to increased regular hearing aid usage and greater access to language input. 2 4 Caregivers may not have the necessary expertise for effective hearing aid management, or they may fail to apply the knowledge. Even when caregivers possess the necessary expertise and confidence for effective hearing aid management, it is well recognized that parents and caregivers do not always adopt the prescribed approach, despite their best intentions to do so. Caregivers may be strongly motivated to ensure that their child wears working hearing aids, but practical techniques to improve potential and provide opportunities for effective hearing aid management may be required. The current study evaluated the hearing aid management experiences of Indian parents of young children. This study included 49 participants, as it was conducted during the peak of the COVID-19 pandemic. All necessary precautions and safety protocols were strictly followed to ensure the well-being of the participants. Despite the small sample size of 49, there were some insights into practical implications and considerations for service providers.

Based on the study's findings, it can be concluded that most parents wanted information and assistance in finding options/accessories for the hearing aids, finding financial assistance for costs, and using a listening stethoscope to determine out when the hearing aid was not working. The study identified two significant findings: parents were unaware that they could receive a loaner hearing aid, and parents were unclear about how to help their child hear better in noisy environments. Munoz et al. 2016 reported conflicting findings as 68% of the parents were aware of the option to receive a loaner hearing aid. 8 This could be a result of the hearing aids being self-sponsored and purchased by the parents and not provided by the Government in India.

The most prevalent challenges identified by parents that influenced their child's hearing aid use were activities (for example, playing, travelling in the car), pressure from others not to use the hearing aids, fear of losing and damaging the hearing aids. In a similar study by Munoz et al. 2015, over 50% of parents reported that activities such as playing outside or riding in the car were a frequent cause of reduced hearing aid use. 6 Munoz et al. 2016 obtained results showing almost a quarter of the parents agreed worry of losing or breaking the hearing aids (20%) was frequent challenge. 8 Caballero et al. 2017 also found fear of losing hearing aids was sometimes a problem for 52% of the parents and always a problem for 14% of the parents. 7

Although most parents were satisfied with the audiologist's support, they wanted the audiologist to be more patient, contact them more frequently after the hearing aids were fitted, and give them more concrete resources (e.g. verbally and in writing). According to Munoz et al. (2016), 27% of English-speaking parents wanted the audiologist to follow up with them more frequently to find out how they were feeling regarding the maintenance of their child's hearing aids. 8 Caballero et al. 2017 reported similar results where 37% of parents in their study wanted to receive concrete resources. 7

## Conclusion

The study aimed to investigate challenges experienced by parents of infants and children with hearing impairment related to skills and confidence with hearing aid management, hearing aid use, and communication with the audiologist from an Indian perspective. Developing a standardized checklist that can be used by professionals when counselling parents of children with hearing aids is a much-needed resource. By addressing parental barriers, working with individuals to develop specific hearing aid management goals, and providing support as needed, professionals may help ensure effective management of hearing aids. Parent-to-parent support, which comes from those who have experienced similar experiences with their children, can help parents become more confident and adept at managing aided hearing. Future studies can focus on conducting out the study on a larger population. An intervention study can be designed to see post-training improvements.
